# Side Chains and the Insufficient Lubrication of Water in Polyacrylamide Hydrogel—A New Insight

**DOI:** 10.3390/polym11111845

**Published:** 2019-11-08

**Authors:** Jincheng Lei, Zidi Zhou, Zishun Liu

**Affiliations:** International Center for Applied Mechanics, State Key Laboratory for Strength and Vibration of Mechanical Structures, Xi’an Jiaotong University, Xi’an 710049, China; jinchenglei@xjtu.edu.cn (J.L.); zhouzidi@stu.xjtu.edu.cn (Z.Z.)

**Keywords:** polyacrylamide hydrogel, side chains, insufficient lubrication of water, Mullins effect

## Abstract

Existing theories cannot predict the mechanical property changes of polyacrylamide hydrogels with different water content because of the absence of side chains. In this study, polyacrylamide hydrogels are prepared and tested to investigate the side chain effect on their mechanical properties. First, the comparison between the effective chain density and total chain density provides proof of the large amount of side chains in the polymer network of PAAm hydrogel. We propose a practical chain density fraction to measure the side chain fraction. Then, the abnormal Young’s moduli-polymer volume fraction relationship reveals that side chains affect the mechanical properties of hydrogel through the insufficient lubrication of water. Water confined in narrow space within a molecular-level size can bear shear force to provide extra deformation resistance. A constitutive mode considering the effect of the insufficient lubrication of water is proposed. Combining this constitutive model with experimental results, we find that this insufficient lubrication of water exists even in equilibrium PAAm hydrogel. Molecular dynamics simulations reveal that this insufficient lubrication of water comes from the constraint of polymer chains. It also demonstrates that when there is insufficient lubrication, the rearrangement of water molecules leads to the persistent energy dissipation in the Mullins effect of PAAm hydrogel.

## 1. Introduction

Monomers solved in water form hydrogels that bond through co-polymerization to the polymeric network with crosslinker molecules using more than two crosslinking sites. The covalent bonds generated by the crosslinking process sustain the 3-dimensional standing structure of hydrogel, while the non-bond interactions retain the surrounding water by hydrogen bonds, electrostatic force, and van der Waals force between the polymer chain and water [1]. Originating from the vast potential applications of hydrogels on biomedicine [2,3] and soft robotics [4,5], the swelling-deswelling process and mechanical behavior of hydrogels have been intensively studied in recent years because of their superior biocompatibility [6] and flexibility [7]. Researchers have proposed a series of constitutive models [8] based on the free energy change resulting from polymer stretching, mixture [9,10], temperature [11,12,13], light [14], ionic concentration [15,16,17,18], and magnetic field [19] to characterize the mechanical behavior and multi-stimuli response of hydrogels [20,21,22,23]. Although the swelling-deswelling deformation of hydrogels has been expounded in many research texts, the change of mechanical properties along with the swelling-deswelling process hardly obtains attention.

In this work, we take polyacrylamide (PAAm) hydrogel as the representative material. PAAm hydrogel is a typical type of single-network hydrogels. Its original and relatively unsophisticated polymer network draws much attention from the hydrogel research community to investigate the microstructure of the crosslinking polymer network and the mechanical behavior. The hyperelastic behavior of polymer network in equilibrium PAAm hydrogel has been well described by Flory-Rehner free energy form [24,25] when imagining an ideal linear chain network. However, the real crosslinking polymer network in PAAm hydrogel is far more complicated than an ideal linear chain network. Side chains, strand entanglement, loop chains, and dangling ends widely exist in a polymer network and have a great influence on the mechanical properties of PAAm hydrogels [26], especially side chains [27]. Fetters et al. [28] propose a backbone dilution degree using the polymerization of polymer backbone and side chains in a polymer network to measure the fraction of side chains in coarse-grain molecular dynamics simulations, while this micro-structural parameter is almost impossible to measure from microscope observations. So far, there is no practical way to quantify the amount of side chains in a polymer network. Existing semi-empirical constitutive models, such as the Flory-Rehner model [24,25] and its improved versions [9,20,22,29], and Langevin chain-based models [10,30], were developed using the statistical averaging of the microstructure effect. Chain density introduced in these models is a practical parameter to track the side chain fraction. The details of this are shown in Section 3.

The semi-empirical constitutive models mentioned above characterize the deformation behavior of the swelling-deswelling process in hydrogels well, whereas they fail to predict the change of mechanical properties along with the change of water content (or polymer fraction). The Young’s modulus-polymer fraction relationship [31,32] first came to our attention in this context. It has been reported that the Young’s modulus of PAAm hydrogel dramatically increases with decreasing water content (or increasing polymer fraction). However, existing constitutive models summarizing the entropy change for polymer stretching by the Neo-Hookean model [29] and Langevin chain model [10,30] seem to not give reasonable predictions. For a uniaxial tension test with $\lambda_{1}{=\lambda ,\lambda }_{2}{=\lambda }^{-\frac{1}{2}}{,\lambda }_{3}{=\lambda }^{-\frac{1}{2}}$ involving incompressible hydrogels, Young’s modulus is extracted in experiments by the initial slope of the nominal stress-stretch curve as follows:

(1) $$E=\frac{\partial }{\partial \lambda }\left(\frac{\partial W_{stretch}}{\partial \lambda }\right)|{}_{\lambda =1}$$

A Neo-Hookean model-based stretch part with $W_{stretch}=\frac{NkT}{2}\left(I_{1}-3\right)$ gives the Young’s modulus $E=3NkT\phi_{p}^{\frac{1}{3}}$, where N is the polymer chain density, kT is the unit thermodynamic energy, and $\phi_{p}$ is the polymer volume fraction. The Langevin chain model-based stretch part with series expansion in an Arruda-Boyce form [33] gives the Young’s modulus $E\propto \phi_{p}^{\frac{1}{3}},\phi_{p}^{-\frac{1}{3}},\ldots ,\phi_{p}^{\frac{1}{3}-\frac{2}{3}n}$. In contrast, pervious experimental results and our experimental results (presented below in this paper) on the $E-\phi_{p}^{n}$ relationship demonstrate that the exponent n is much higher than $\frac{1}{3}$ in theory. One interpretation of this phenomenon is that the increasing concentration of the polymer network makes hydrogel change from a diluted solution to a semi-diluted or even a fully concentrated solution, leading to the polymer chain conformation distribution gradually deviating from a Gaussian distribution or Langevin distribution. Thus the entropy change of the polymer network stretching should be modified. However, this interpretation neglects the contribution of side chains. The evolution of shear modulus with decreasing water content influences the dramatically increasing effect of side chains. Despite this, researchers still do not know how side chains provide so much deformation resistance.

The Mullins effect in PAAm hydrogel provides another example which existing theory fails to predict. A prominent Mullins effect in double network hydrogels has been observed experimentally [34] and ascribed to the effect of secondary dissipation polymer chains and sacrificial ionic bonds [35]. But for a simpler neutral polymer network, a polyacrylamide (PAAm) hydrogel without additional dissipation polymer chains also has the Mullins effect [36]. Bai et al. [37,38] found that PAAm hydrogels underwent persistent energy dissipation, even after thousands of cycling loading events, which apparently can be no longer attributed to chain scission. Moreover, our experiments (presented below in this paper) show that this Mullins effect is enhanced with decreasing water content. It is apparent that both the Young’s modulus and the Mullins effect in PAAm hydrogels deviate from the theoretical predictions purely because of the change of water content, while the effect of the solvent water is not addressed in existing constitutive equations. Therefore, in addition to the crosslinking polymer network and the polymer network-water mixture, we propose that side chains play quite an important role in the mechanical behaviors of PAAm hydrogels due to the insufficient lubrication of water, which was ignored in previous research.

In order to clarify the side chain effect and the insufficient lubrication of water in hydrogel, we prepared PAAm hydrogels with varying water content, conducted uniaxial tension tests and cycling loading tests (Section 2) to measure the fraction of side chains, and analyzed the insufficient lubrication of water in PAAm hydrogel. In Section 3, a new constitutive model is proposed to quantify the lubrication of water in PAAm hydrogels. Molecular dynamics simulations are carried out to reveal the microscopic mechanism of the lubrication of water and explain the Mullins effect in PAAm hydrogels. We hope this study can give insights into the microstructure of hydrogels and its impact on the mechanical properties.

## 2. Materials and Mechanical Tests

Two types of PAAm hydrogels with different precursor ratio were prepared. The first type of PAAm hydrogels were prepared with the following substances: Acrylamide (AAm, Aladdin Industrial Corp., Shanghai, China), N,N-methylenebisacrylamide (MBAA, Aladdin Industrial Corp., Shanghai, China), ammonium persulfate (APS, Aladdin Industrial Corp., Shanghai, China), tetramethylethylenediamine (TEMED, Keshi, Chengdu, China), and water were used as a monomer, crosslinker, initiator, accelerator, and solvent with a weight ratio of 1:0.001:0.001:0.004:3.994. The second type of hydrogel uses the weight ratio of a precursor with 1:0.0022:0.0019:0.004:3.340. The precursor solution was stirred sufficiently and then injected into glass molds (10 mm × 10 mm × 1 mm) to form a gel at room temperature for 1 day. The as-prepared PAAm hydrogels were immersed into deionized water to reach an equilibrium state for 1 day. Then PAAm hydrogels were dehydrated by exposure in air for different times and then placed in sealing bags for half a day to achieve a homogenous water distribution. The water content of each hydrogel sample was obtained by the current mass compared to the original solution mass of this sample. PAAm hydrogels with different water contents were then cut into dumbbell-shaped standard samples for uniaxial tension tests. A stretch machine (SHIMADZU AGS-X, Shimadzu Corp., Kyoto, Japan) provided a different loading rate in the range of 10–300 mm/min and output force-displacement data, which were then converted to nominal stress-stretch curve manually.

**Figure 1:**
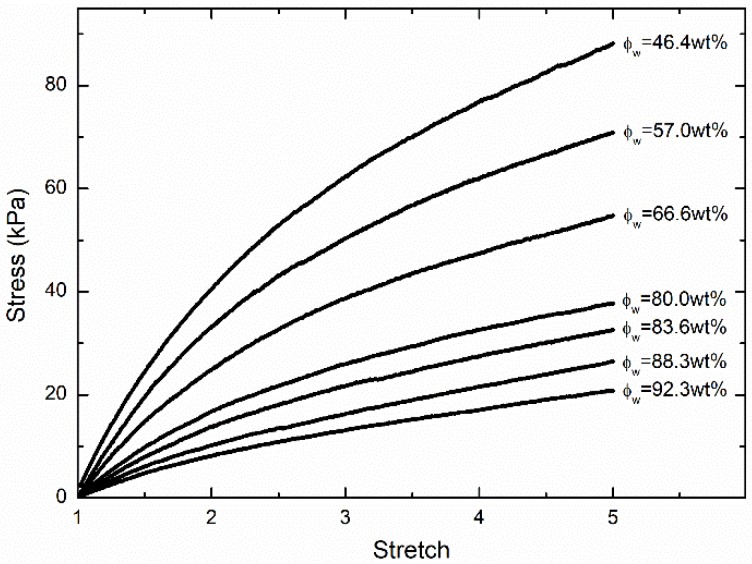
Nominal stress-stretch curve for hydrogels with different water content levels.

**Figure 2:**
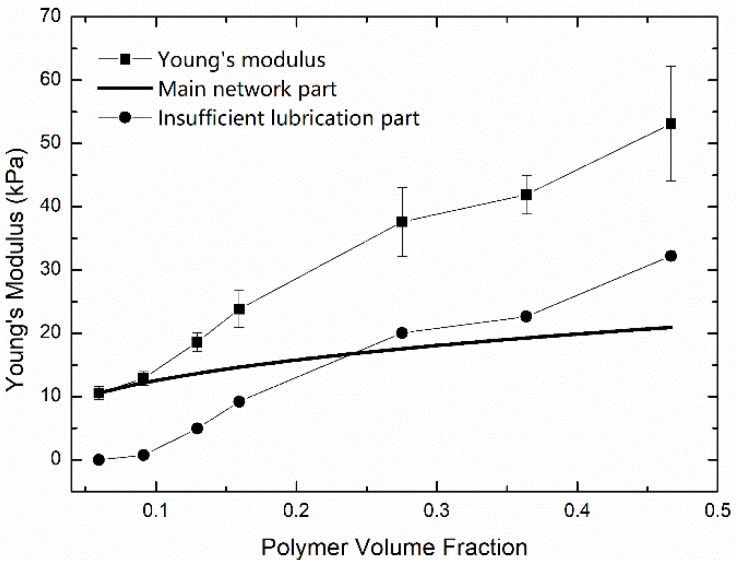
Young’s moduli of PAAm hydrogels with different polymer volume fractions (Square dots with error bars are Young’s moduli of PAAm hydrogel with different water content. Assuming the negligible effect of side chains in equilibrium PAAm hydrogel, solid line shows the Young’s moduli contributed by the main network, while circle dots are the rest of Young’s moduli contributed by insufficient lubrication of water).

Figure 1 shows the nominal stress-stretch curve at the stretch rate of 1/min for the first type of hydrogels with water content $\phi_{w}=$ 92.3 wt%, 88.3 wt%, 83.6 wt%, 80 wt%, 66.6 wt%, 57.0 wt%, and 46.4 wt%, respectively. The Young’s moduli of hydrogels with different water contents were extracted by fitting the initial part of the nominal stress-stretch curve as the black dots shown in Figure 2. Three independent uniaxial tension tests with the loading rate of 1/min were carried out for hydrogels with a certain water content. Obviously, the Young’s modulus-polymer volume fraction relationship shows a power-law relationship with any exponent much larger than $\frac{1}{3}$.

**Figure 3:**
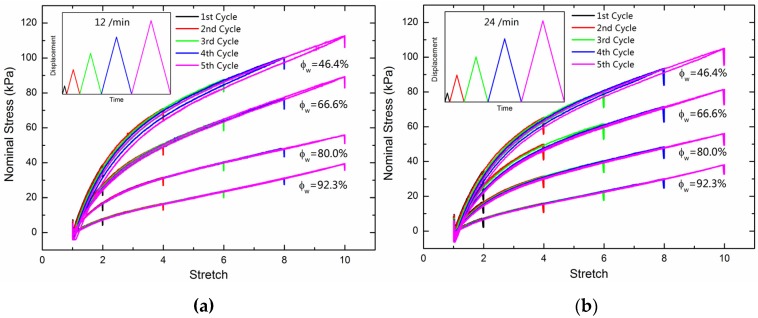
(**a**) Nominal stress-stretch curves for the cycling loading of PAAm hydrogels with different water content at the loading rate of 12/min; (**b**) Nominal stress-stretch curves for the cycling loading of PAAm hydrogels with different water contents at the loading rate of 24/min.

We further investigate the Mullins effect of PAAm hydrogels with water content 92.3 wt%, 80.0 wt%, 66.6 wt%, and 46.4 wt%. Uniaxial cycling loading was imposed on one sample to provide a maximum stretch of 2, 4, 6, 8, and 10/min in turn. We adopted two loading rates of 12/min and 24/min for different samples. Figure 3a,b show the nominal stress-stretch curves of the cycling loading with loading rates of 12/min and 24/min, respectively. The Mullins effect became more visible with a decreasing water content. Higher loading rate does not recognize a larger stress-stretch hysteresis loop.

## 3. Results and Discussions

### 3.1. Side Chains

Chain density is the structural property that we can directly extract from the shear modulus of PAAm hydrogel. Here a practical measurement of side chain fraction was proposed by comparing the chain density calculated from precursor ratio and extracted from mechanical tests. Two types of equilibrium PAAm hydrogels with the precursor ratio AAm:MBAA=1:0.001 and 1:0.0022 are prepared. The water contents are 92.3 wt% and 93 wt%, respectively. If we assume that all cross-linkers participate in polymerization, the total chain density in dry state can be estimated from the precursor ratio by

(2) $$N_{total}=2\frac{m_{MBAA}}{M_{MBAA}}\cdot \frac{1}{V_{dry}}$$

where $m_{MBAA}$ is the total mass of crosslinker MBAA, $M_{MBAA}$ is the mass of one MBAA molecule, i.e., $2.57\times 10^{-22}$ g, and $V_{dry}$ is the total volume of dry polymer. Each MBAA provides four crosslinking points and possesses two polymer chains. Since the polymerization has almost completed, this chain density is reliable.

On the other hand, the chain density can also be extracted from the Young’s modulus in mechanical tests. Three independent uniaxial tension tests are carried out for samples of each type of equilibrium hydrogel. Theoretically, Young’s modulus of hydrogels can be derived from the well-established constitutive models. For equilibrium hydrogels, the free energy form is

(3) $$W=W_{stretch}+W_{mix}$$

which provides unambiguous physical meaning for this polymer network-water system, which in turn has been expounded in abundant references [9,20,29]. Given that the bulk modulus of equilibrium hydrogel is about four orders of magnitude higher than the shear modulus, hydrogel is considered to be a nearly incompressible material. This makes only the stretch part of the free energy $W_{Stretch}$ contribute to the deformation resistance. The most widely used Neo-Hookean-based stretch part with

(4) $$W_{stretch}=\frac{1}{2}NkT\left(J_{0}^{\frac{2}{3}}I_{1}-3\right)$$

leads to the Young’s modulus of hydrogel with

(5) $$E=3NkT\phi_{p}^{\frac{1}{3}}$$

by substituting Equation (4) into Equation (1). This elegant expression connects the microscopic structural properties with the macroscopic mechanical properties of hydrogel. Here the effective chain density N represents the density of polymer chains which constitutes the main polymer network of PAAm hydrogel. It is obtained from the Young’s modulus of equilibrium PAAm hydrogel.

**Figure 4:**
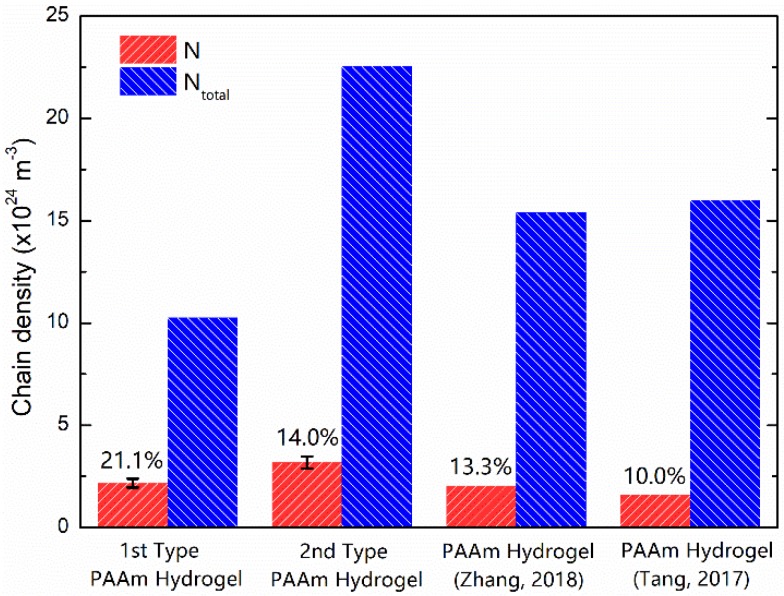
Extracted chain density vs. estimated chain density for three types of PAAm hydrogels.

Figure 4 gives comparison between N and $N_{total}$ in our experiments and others’ experiments [31,39]. Obviously, all the total chain densities are much larger than the effective chain density. If we use this chain number ratio $N/N_{total}$ to evaluate the number fraction of main network, only 21.1%, 14.0%, 13.3%, and 10.0% of the chains contribute Young’s moduli of the two type of PAAm hydrogels in our experiments and PAAm hydrogels in others’ experiments, respectively. In fact, the conformation of polymer chains in hydrogels is far more complicated beyond the description of chain density in a statistical way. The effective chain density may be slightly affected by the random chain length, which is denoted as a network imperfection [36]. Meanwhile, the different polymerization degree of main network and side chains, which may be affected by the dosage of an initiator, accelerator and solvent, also affect the mass fraction of side chains. Yet the conspicuous discrepancy here convinces us of the large amount of side chains in the polymer network.

**Figure 5:**
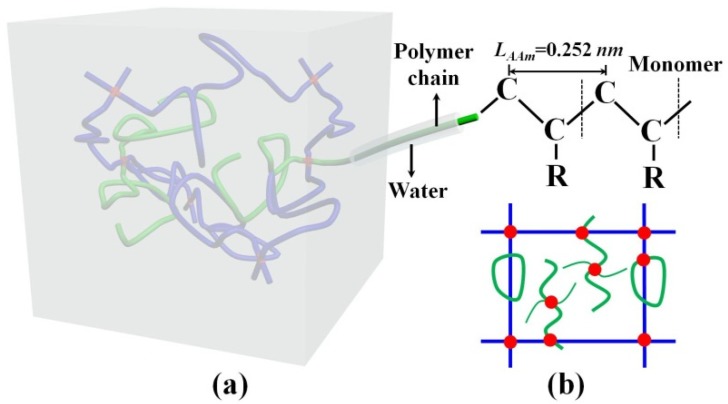
(**a**) Schematic diagram of the polymer network-water structure of PAAm hydrogel. (**b**) Schematic diagram of the main polymer network and the dangling chains (Blue chains represent the main network; Green chains are the dangling chains; Red joints are the crosslinkers).

Side chains are often those polymer chains whose two ends are attached to the main network at the same site (the green loop attached to main network in Figure 5b) or one of two ends is free (green chain attached to main network in Figure 5b). For some cases, a side chain connected with one MBAA with two ends forms a loop and may trap several chains in main network, or a self-connected side chain forms a loop and may trap several chains in the main network. Despite these specific molecular defects in the polymer network, we denote side chains as those polymer chains which do not contribute to the deformation resistance. With the understanding of side chains, the mean chain length in real PAAm hydrogel cannot be estimated from the ideal linear chain network. Hence, two chain densities N and $N_{total}$ actually describe polymer chains with different degrees of polymerization. The parameter $N/N_{total}$ underestimates the real ratio of the main network in PAAm hydrogel, whereas it is still a practical parameter for experimentally evaluating the amount of main network chains and side chains.

### 3.2. Insufficient Lubrication of Water

Although we determined the large amount of side chains in hydrogels, how this outcome affects the Young’s modulus-polymer fraction relationship and the Mullins effect is still not clear. Since both Young’s modulus and Mullins effect are related to water content, we ascribe the side chain effect to the insufficient lubrication of water. Figure 5a gives a schematic diagram of the microstructure of PAAm hydrogel. Considering that hydrogen bonds formed between the polymer-water interface are stronger than the van der Waals force between polymer chains, the structure of hydrogel can be regarded as the polymer threads wrapped by water layer. Polymer chains in hydrogels interact with each other by a non-bond weak force through water. During the deformation of hydrogel, since PAAm hydrogel is nearly incompressible, the deformation comes from the relative slip between adjacent polymer chains, where water plays a role of a lubricant to facilitate the relative movement. However, this lubrication of water is always insufficient.

To quantify the lubrication of water, it is necessary to analyze the mean water thickness between polymer chains in PAAm hydrogel. During the deformation of PAAm hydrogel, main network undergoes stretching or contracting, and all chains undergo relative slip with adjacent chains at the same time. If we take the hydrogel as cylindrical polymer chains wrapped by water layer as illustrated in Figure 5a, the thickness of the water layer can be derived from the water content. Since polyacrylamide is formed by the co-polymerization through ethylenic bonds in AAms, each AAm can be considered as a cylinder with the height of the projection length being two carbon-carbon bonds, i.e., $L_{AAm}=0.252$ nm. Then the total length of polymer chains per unit volume can be expressed by

(6) $$L_{chain}=\frac{\phi_{p}\rho_{AAm}L_{AAm}}{M_{AAm}}$$

where $\phi_{p}$ is the polymer volume fraction of the hydrogel, $\rho_{AAm}$ is the density of AAm 1.32 g/cm^3^, and $M_{AAm}$ is the mass of one AAm molecule at $1.18\times 10^{-22}$ g. The mass of crosslinker MBAA is negligible here. Thus, the mean water layer thickness between two adjacent polymer chains can be estimated by twice the difference between the mean radius of chain-water cylinder and the chain cylinder as follows:

(7) $$d=2\sqrt{\frac{M_{AAm}}{\pi \rho_{AAm}L_{AAm}}}\left(\frac{1}{\sqrt{\phi_{p}}}-1\right).$$

**Figure 6:**
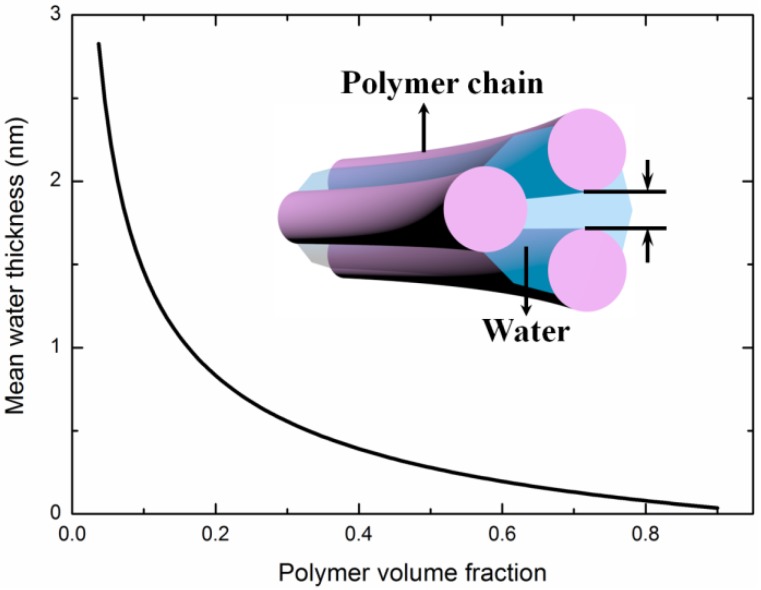
Mean water thickness of PAAm hydrogels with different water content.

Using Equation (7), the water layer thickness of PAAm hydrogel is shown in Figure 6 with respect to the water content. It can be seen that the mean water layer thickness is in a sub-nanometer level when the water content is below 84%. Does the lubrication of water layer still work in such a narrow space? Previous researchers in hydrodynamics have demonstrated that when water is confined in a space with the characteristic length being smaller than 3–5 times the diameter of water molecule (~0.3 nm), water shows dramatic increase of viscosity for many orders of magnitude and even undergoes a liquid-to-solid transformation [40,41,42]. In other words, a highly confined water layer can bear shear force, which results in the insufficient lubrication of water in PAAm hydrogel. This shear force emerges to be the dominant parts of the resistance against deformation in a hydrogel with low water content.

The direct performance of the insufficient lubrication of water is the extra Young’s modulus, as shown in Figure 2. Since the crosslinked polymer network in a PAAm hydrogel has been established during the gelation process, the effective chain density N is supposed to be constant during the swelling-deswelling process. We assumed that the effect of insufficient lubrication of water in equilibrium PAAm hydrogel can be neglected and extracted the chain density for the main network from equilibrium hydrogel using Equation (5). The blue line in Figure 2 gives the Young’s modulus of PAAm hydrogel scaling with the volume change based on Equation (5). It is obvious that theoretical predictions using Equation (5) miss the part of Young’s modulus induced by the insufficient lubrication of water, as the circle dots show in Figure 2. The rapid increasing of the extra Young’s modulus is caused by the highly confined water layer between polymer chains. In an energetic view, the free energy in equilibrium PAAm hydrogel is dominated by the entropy of main network, while in a PAAm hydrogel with low water content, the deformation energy of side chains becomes dominant.

### 3.3. Constitutive Model with Insufficient Lubrication of Water

In this section, a constitutive model to take into account the insufficient lubrication of water is proposed. Taking the dry polymer as the reference state, the deformation gradient of hydrogel can be decomposed into volumetric deformation and shape change deformation by a multiplicative decomposition as follows

(8) $$F=F_{v}F_{s}$$

where $F_{v}$ is the volumetric deformation gradient caused by free-swelling with $J_{0}=\det\left(F_{v}\right)$ and $F_{s}$ is the shape change deformation gradient with $\det\left(F_{s}\right)=1$ based on incompressible assumption. Since water bears shear force during the shape deformation, our free energy form for hydrogels can be expressed as

(9) $$W\left(F_{v},F_{s}\right)=W_{stretch}\left(F_{v},F_{s}\right)+W_{mix}\left(F_{v}\right)+W_{lub}\left(F_{s}\right)$$

where $W_{stretch}$ is the entropy of stretching polymer network, $W_{mix}$ is the entropy and enthalpy for mixing polymer and water, and $W_{lub}$ is the shear deformation energy induced by the insufficient lubrication of water. The Flory-Rehner free energy form is adopted as the first two terms in Equation (9) as follows

(10) $$W_{stretch}=\frac{NkT}{2J_{0}}\left(J_{0}^{\frac{2}{3}}{\bar{I}}_{1}-3\right),W_{mix}=\frac{kT}{J_{0}}\left(\frac{J_{0}-1}{v}\ln\frac{J_{0}-1}{J_{0}}+N\ln\frac{1}{J_{0}}-\frac{1}{v}\frac{\chi }{J_{0}}\right)$$

where v is the volume of the water molecules and χ is the dimensionless Flory interaction parameter which characterizes the interaction between polymer and water. We choose the shear deformation energy term to be a simple form as

(11) $$W_{lub}=G_{lub}{\bar{I}}_{2}$$

where ${\bar{I}}_{2}$ is the second invariant for the right Cauchy-Green strain tensor of shape deformation. ${\bar{I}}_{2}$ integrates the deviatoric strain which microscopically embodies the slippage between adjacent polymer chains. $G_{lub}$ is defined as the lubrication modulus accounting for the microscopic lubrication of water. With sufficient lubrication of water, the slip force is not comparable to the force induced by a stretching polymer network in equilibrium hydrogels. The lubrication modulus diminishes to negligible levels in hydrogels with high water content. In contrast, the lubrication modulus increases in hydrogels with lower water content because of insufficient lubrication. It should be noted that this constitutive model does not include any time-dependent term to characterize the viscoelasticity. Although a minor Mullins effect is observed in experiments, the following molecular dynamics simulations in the next section prove that this Mullins effect is not caused by viscoelasticity.

When hydrogel is under a uniaxial tension test, the loading condition with $\lambda_{1}=\lambda ,\lambda_{2}=\lambda^{-\frac{1}{2}},\lambda_{3}=\lambda^{-\frac{1}{2}}$ is adopted due to the near incompressibility of hydrogel. The hydrostatic force term induced by chemical potential difference is eliminated by the boundary condition with the nominal stress $P_{22}=P_{33}=0$. The free energy form of hydrogel leads to the nominal stress-stretch relation along the loading direction as follows

(12) $$P_{11}=\frac{\partial W}{\partial \lambda }=NkT\phi_{p}^{\frac{1}{3}}\left(\lambda -\lambda^{-2}\right)+2G\left(1-\lambda^{-3}\right)$$

Meanwhile, the Young’s modulus can be expressed by substituting free energy into Equation (1) as

(13) $$E=3NkT\phi_{p}^{\frac{1}{3}}+6G.$$

This equation gives a clear definition of the lubrication modulus and provides a simple way to access the microscopic lubrication evolution during swelling-deswelling process.

**Figure 7:**
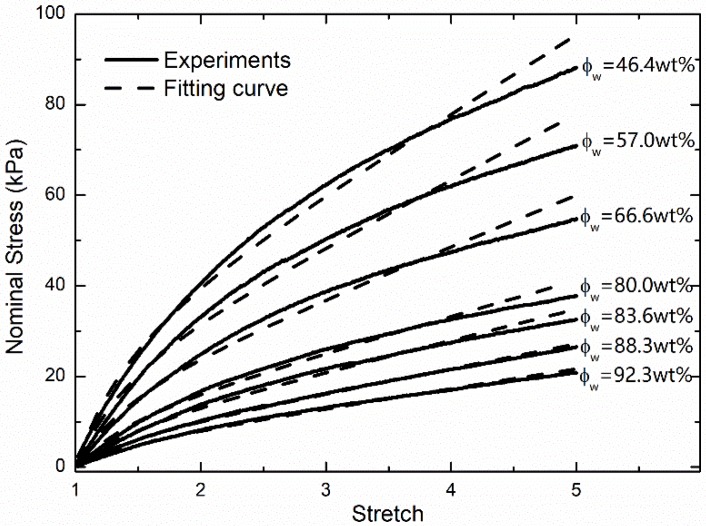
Fitting the nominal stress-stretch curves in our experiments.

**Figure 8:**
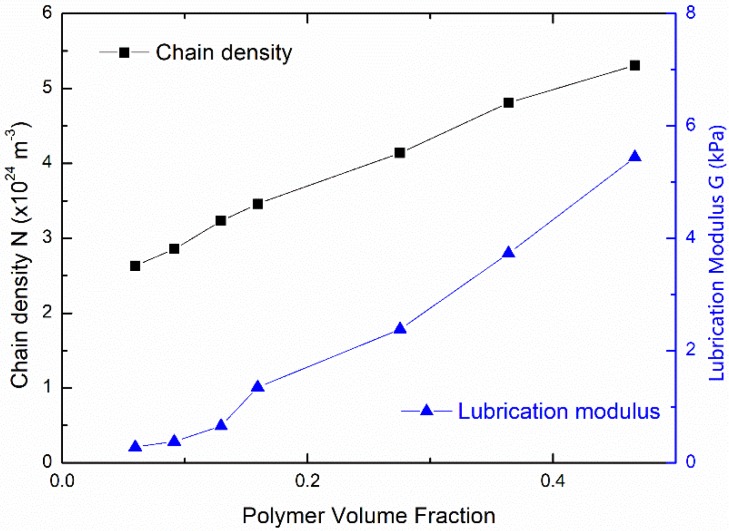
Two fitting parameters vs. the polymer volume fraction (Black dots are chain density N; Blue dots are lubrication modulus G).

With this constitutive model, we further fitted the initial parts of the nominal stress-stretch curves in Figure 1 using Equation (12) with fitting parameters N and $G_{lub}$. Fitting stress-stretch curves are shown in Figure 7. The fitting quality tends to increase with an increasing water content. Two fitting parameters for PAAm hydrogels with different water content are shown in Figure 8. It can be seen that the obtained lubrication modulus goes up with a decreasing water content, which is the same trend as for the previous results in Figure 2. Meanwhile, it shows that even the equilibrium hydrogel has considerable lubrication modulus 280 Pa, i.e., about 16.0% of the Young’s modulus. This reveals the more ubiquitously insufficient lubrication of water in PAAm hydrogels with any water content. It also implies that the main network fraction is even lower than what we obtained in Figure 4. We can also find that the obtained chain density goes up with a decreasing water content. This is not consistent with our assumption that the chain density of main network remains constant during the swelling-deswelling process.

### 3.4. Mullins Effect

Recalling the Mullins effects in double network hydrogels, a large amount of deformation energy during cycling loading was dissipated by the secondary chains and sacrificial ionic bonds, while PAAm hydrogels kept dissipating a small amount of energy, even after thousands of cycles [37,38]. We infer that this persistent energy dissipation was also caused by the insufficient lubrication of water. PAAm hydrogels with lower water content recognize a more visible Mullins effect as shown in Figure 3a,b. Similarly, the energy dissipation in PAAm hydrogels results from the side chains and the insufficient lubrication of water.

**Figure 9:**
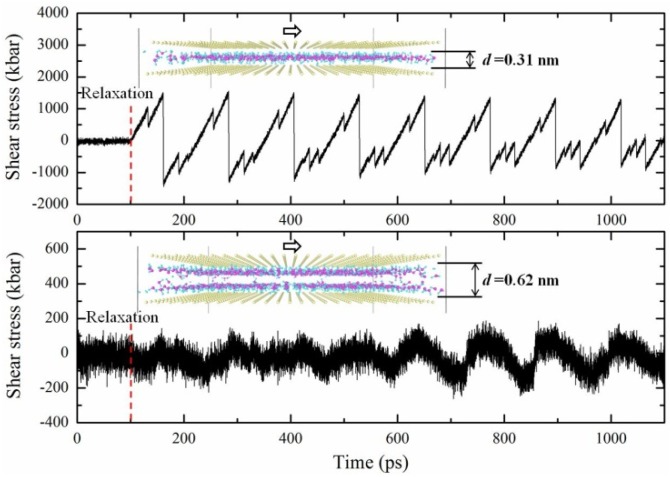
Shear stress of water vs. time during the slip of the upper boundary (Insets are the atomic models for molecular dynamics. Yellow oxygen atoms form upper and lower boundaries in atomic models. Cyan hydrogen atoms and magenta oxygen atoms compose water molecules. The model in the upper figure has one layer of water molecules with the thickness 0.31 nm. The model in the lower figure has two layers of water molecules with the thickness 0.62 nm).

Side chains tangle in hydrogel. Unlike the main network, the free ends of side chains can easily drift away. During the cycling loading, it is very easy for side chains to achieve new chain conformation because of a chain stretch or contraction. In PAAm hydrogels with low water content, side chains tend to get stuck due to a lack of restoring force. Here the energy dissipation occurs and can be ascribed to the conformation entropy change of side chains. When side chains find another energetic favorable conformation and get stuck, fatigue occurs. Fatigue during the cycling loading of hydrogels comes from the accumulation of both chain scission and the dissociation of side chains. Apparently, it is not the reason for the persistent energy dissipation in PAAm hydrogel because the dissipated energy in thousands of cycles is far larger than the deformation energy. In order to understand the persistent energy dissipation, atomic models for a thin water layer [43] were constructed for molecular dynamics simulations to realize the lubrication of water. Two atomic models as shown in insets in Figure 9 are composed of upper and lower boundaries of oxygen atoms and water molecules inside. Boundary oxygen atoms are in square array to represent the hydrogen-bonding sites for water, corresponding to the oxygen atoms in amide groups of AAm molecules. Since water molecules show a layered arrangement when confined in a molecular narrow space, one layer of water molecules with a thickness of 0.31 nm was inserted into the first model and two layers of water molecules with a thickness of 0.62 nm were inserted into the second model. LAMMPS [44] was used to conduct all the molecular dynamics simulations. Tip4p force field [45] was adopted to characterize the interaction of water molecules and boundary oxygen atoms in a canonical (NVT) ensemble. After the relaxation for 100 ps, the slip simulation was carried out by moving the upper boundary along a certain in-plane direction for another 1000 ps. The shear stress of water vs. time curves is shown in Figure 9. It is very clear that water molecules bear the shear force periodically with the period of the lattice constant of boundary atoms. This resulted from the periodic potential field caused by the boundary atom arrays. Work done by the shear force was dissipated by the rearrangement of layered water molecules. The more confined water layer in the first model was much harder to accomplish this rearrangement than that in the second model, which was caused by having less space and manifested as the much larger force water bears in the first model.

The qualitative results in molecular dynamics simulations correspond directly to the mechanical properties of hydrogels. Larger thickness of water layer represents higher water content in hydrogels as shown in Figure 6. The corresponding lower force water bears leads to a lower lubrication modulus. Work dissipation in the rearrangement of water molecules during the boundary slip induces the minor persistent energy dissipation during cycling loading, which is different from viscoelasticity. These molecular dynamics simulations provide a microscopic view on the mechanical behavior of hydrogels. It can be concluded that the insufficient lubrication of water resulted from the highly confined interaction between polymer chains. The molecular scale water thickness in PAAm hydrogel demonstrated insufficient lubrication.

## 4. Conclusions

In this study, PAAm hydrogel samples were prepared and tested by uniaxial tension tests and cycling tests to investigate the mechanical properties and give insight into the microstructure of hydrogels. First, we compared the chain density calculated from the precursor ratio in preparation and the chain density extracted from the Young’s modulus of prepared equilibrium hydrogel samples. The conspicuous discrepancy of two chain density indicated the large amount of side chains in the polymer network. A practical parameter $N/N_{total}$ was proposed to evaluate the main network fraction of the polymer network in a hydrogel. A large amount of side chains provided increasing Young’s modulus for PAAm hydrogels with a decreasing water content. We ascribed this side chain effect to the insufficient lubrication of water, which is directly related to the molecular-level water thickness between adjacent polymer chains. In order to quantify the insufficient lubrication of water, a constitutive model was proposed by considering the free energy caused by the relative slip between polymer chains. A concise lubrication modulus was defined to characterize the lubrication of water in different hydrogels with different water content levels. By fitting the nominal stress-stretch curves in experiments using this constitutive model, it shows that the insufficient lubrication of water exists in PAAm hydrogels with all water content levels. The results from molecular dynamics simulations support this conclusion. Upon knowing the large amount of side chains and the ubiquitously insufficient lubrication of water, the persistent energy dissipation in Mullins effect of the PAAm hydrogel was ascribed to the rearrangement of water molecules. By learning from the PAAm hydrogel, we hope this study can deepen understanding of the microstructure of the polymer chains in hydrogels and its influence on mechanical behavior.
